# Evaluating Functional Diversity: Missing Trait Data and the Importance of Species Abundance Structure and Data Transformation

**DOI:** 10.1371/journal.pone.0149270

**Published:** 2016-02-16

**Authors:** Maria Májeková, Taavi Paal, Nichola S. Plowman, Michala Bryndová, Liis Kasari, Anna Norberg, Matthias Weiss, Tom R. Bishop, Sarah H. Luke, Katerina Sam, Yoann Le Bagousse-Pinguet, Jan Lepš, Lars Götzenberger, Francesco de Bello

**Affiliations:** 1 Department of Soil Science, Faculty of Natural Sciences, Comenius University, Bratislava, Slovak Republic; 2 Department of Botany, Faculty of Science, University of South Bohemia, České Budějovice, Czech Republic; 3 Institute of Ecology and Earth Sciences, University of Tartu, Tartu, Estonia; 4 Institute of Entomology, Biology Centre CAS, České Budějovice, Czech Republic; 5 Department of Zoology, Faculty of Science, University of South Bohemia, České Budějovice, Czech Republic; 6 Institute of Soil Biology, Biology Centre CAS, České Budějovice, Czech Republic; 7 Department of Ecosystem Biology, Faculty of Science, University of South Bohemia, České Budějovice, Czech Republic; 8 Department of Biosciences, University of Helsinki, Helsinki, Finland; 9 Department of Earth, Ocean and Ecological Sciences, University of Liverpool, Liverpool, United Kingdom; 10 Centre for Invasion Biology, Department of Zoology and Entomology, University of Pretoria, Pretoria, South Africa; 11 School of Biological Sciences, University of East Anglia, Norwich, United Kingdom; 12 Department of Zoology, University of Cambridge, Cambridge, United Kingdom; 13 Area de Biodiversidad y Conservación, Departamento de Ciencias, Escuela Superior de Ciencias Experimentales y Tecnología, Universidad Rey Juan Carlos, C/ Tulipán s/n, Móstoles, Spain; 14 Institute of Botany, Biology Centre CAS, Třeboň, Czech Republic; University of Sydney, AUSTRALIA

## Abstract

Functional diversity (FD) is an important component of biodiversity that quantifies the difference in functional traits between organisms. However, FD studies are often limited by the availability of trait data and FD indices are sensitive to data gaps. The distribution of species abundance and trait data, and its transformation, may further affect the accuracy of indices when data is incomplete. Using an existing approach, we simulated the effects of missing trait data by gradually removing data from a plant, an ant and a bird community dataset (12, 59, and 8 plots containing 62, 297 and 238 species respectively). We ranked plots by FD values calculated from full datasets and then from our increasingly incomplete datasets and compared the ranking between the original and virtually reduced datasets to assess the accuracy of FD indices when used on datasets with increasingly missing data. Finally, we tested the accuracy of FD indices with and without data transformation, and the effect of missing trait data per plot or per the whole pool of species. FD indices became less accurate as the amount of missing data increased, with the loss of accuracy depending on the index. But, where transformation improved the normality of the trait data, FD values from incomplete datasets were more accurate than before transformation. The distribution of data and its transformation are therefore as important as data completeness and can even mitigate the effect of missing data. Since the effect of missing trait values pool-wise or plot-wise depends on the data distribution, the method should be decided case by case. Data distribution and data transformation should be given more careful consideration when designing, analysing and interpreting FD studies, especially where trait data are missing. To this end, we provide the R package “traitor” to facilitate assessments of missing trait data.

## Introduction

Functional trait-based approaches are increasingly used in ecology for understanding the environmental and evolutionary processes underlying biological diversity [1,2]. While traditional measures of biodiversity encompass the richness and abundance of organisms in an ecosystem, trait-based studies can provide additional information on their functions. Where taxonomic diversity categorises organisms by relatedness, functional diversity (FD) captures the extent of the differences between organisms in terms of functional traits, i.e. the measurable characteristics associated with their fitness and ecological function [3]. A functional approach can therefore allow generalizations beyond taxa and biogeographical regions, and can reveal both how species coexist together and how they might affect multiple ecosystem processes [4,5].

Functional diversity can be measured with different FD indices, which capture different aspects of diversity: functional richness, functional evenness and functional divergence (for full review see [6] and references therein). All indices are calculated using both trait and species composition data. Intuitively, the more species for which trait data are available, the more FD indices will reflect the real community values [7]. However, complete trait data is often not available for all species because it might be difficult to measure certain traits for particular species, time and resources are usually limited, and species might be very rare. It is particularly common to be missing trait data for rare species, and they are generally the first to be omitted in incomplete datasets [7]. The omission of the rare species first is mainly because the most abundant species are expected to have the most functional influence on ecosystem functioning (see 'mass ratio hypothesis'[8] and [9]) and are therefore sampled with higher priority.

It also remains unclear what proportion of species one needs to measure to make a reliable assessment of functional diversity, and whether it is possible to generalize this across study systems, different taxa or different sampling methods. This is an increasingly important issue to address with the advent of trait databases, of which even the largest are still plagued by missing data [10] reducing their power for larger-scale comparative studies. Many trait-based studies use an 80% relative abundance threshold as a rule of thumb, i.e. sampling trait information for the most common species that comprise at least 80% of the total abundance in a community [11]. This measure was proposed for and is adequate for community weighted mean of traits (CWM), which is not sensitive to outliers and focuses on the most dominant species in a community [9]. In contrast to CWM, FD indices such as functional richness, evenness and divergence encompass the variability of both rare and dominant species and are therefore more sensitive to missing trait information [7]. In general FD values decline in reliability with missing trait data [6], however, how this decline affects the biological conclusions drawn from such indices still remains to be tested.

Since FD indices are sensitive to missing trait data, it is important to make an *a priori* decision on how to deal with potential data gaps. One approach is to fill in the missing trait data by imputation [12,13]. Another option is to set ‘safe’ trait data completeness thresholds, i.e. the minimum proportion of species for which trait data must be obtained, usually prioritizing the most dominant [11]. Data completeness thresholds can be applied either on the whole assessed pool of species or in each plot separately [7]. The choice of applying either a pool-wise or plot-wise threshold also influences the way researchers conduct trait sampling, and depends on whether the focus is on species abundant in the whole dataset (pool-wise) or locally (plot-wise) [14], as well as on the rate of species turnover between plots and/or habitat heterogeneity (ie. beta diversity). For example, along ecological gradients with high replacement of dominants across locations, setting the trait sampling thresholds for each plot can reveal more relevant information than setting it for the whole pool [15].

FD indices are calculated using both species composition and trait data, therefore the structure and the choice of transformation of both types of data can strongly influence their computation. Abundance structure reflects the dominance patterns of a community [16,17], with the most abundant species expected to have the most functional influence (see 'mass ratio hypothesis'[8]). Abundance data can spread over several orders of magnitude [18–20]. Therefore, abundance data are often transformed to avoid relatively small differences between species, which are often biologically relevant, being overshadowed by larger ones. Trait data present the same problem, with traits varying on different orders of magnitude, but with small biological differences at low values of a trait being often as biologically relevant as bigger differences at greater values. As such, traits can also be transformed, which can influence the measures of functional differences between species [20]. For example, traits related to size or weight are mostly skewed in distribution and thus routinely transformed to meet normality criteria. In such cases, log-scaled values rather than absolute values may better reflect the study system in question [21,22]. Both abundance and trait transformation changes the relative influence of rare species as well as affecting features of the distribution (namely variance, skewness and kurtosis), and effectively decreases the differences between rare and abundant species. In synthesis, both species abundance and trait structure and data transformation could affect the resulting FD indices and their interpretation. Although some studies have been conducted on the effect of missing data [7,11,12,23], the additional effects of abundance and trait structure and data transformation on missing trait data are not yet resolved. This is a crucial issue to address, since FD indices of communities with different trait and abundance distributions may differ in their sensitivity to the same amount of missing data and may not be directly comparable.

Here we test the specific questions: (1) How do missing trait data influence the robustness and reliability of FD indices; (2) How does defining a safety threshold for missing trait data either pool-wise or plot-wise influence the robustness of FD indices to missing trait data; (3) How do abundance structure, abundance measures and abundance transformation affect the robustness of FD indices to missing trait data; (4) How do trait distribution and transformation affect the robustness of FD indices to missing trait data?

## Material and Methods

### Datasets

We selected three datasets that represent (1) different groups of organisms (i.e. plants, invertebrates, and vertebrates), and for which we have complete information on species abundance and trait data; (2) different types of abundance sampling methods; (3) different dominance-diversity structure. For each of the datasets we selected traits that were available for all sampled species, are commonly used, and represent important dimensions of species ecological strategies([2,22,24] and references therein). These traits represented both continuous and categorical traits. More details on all study systems and traits are given in S1 Appendix. Taking the lead from previous studies on the effects of missing data [6, 7, 8, 19] we focus on real communities that reflect the nuances that are important for the structuring of species assemblages. At the same time, to expand upon previous work, we decided to test different types of communities and sampling patterns to assess consistency in the patterns detected.

Plant data were collected from an oligotrophic species-rich wet meadow in the south-western Czech Republic with 12 plots differing in their management [25,26]. Three methods of measuring plant abundance were adopted: (1) species frequency in quadrats in each plot (henceforth "frequency"), (2) percentage cover estimates performed visually from the centre of each plot (henceforth "cover"), and (3) biomass, where plants were clipped, sorted into species, oven-dried and weighed. We assessed two categorical (growth form and position of leaves along the stem) and three continuous traits (canopy height, specific leaf area and seed mass) of 62 species.

Ant data were collected at the Stability of Altered Forest Ecosystems (SAFE) project in Sabah, Malaysia [27,28]. In total the survey included 59 plots in different habitat types; oil palm, logged forest, and old growth forest. Ants were hand collected from soil pits and dead wood in quadrats in each survey point. The abundance of each species was expressed as number of individuals. We assessed two categorical (pilosity and sculpturing) and two continuous traits (head length and the ratio of leg length to body size) of 297 species.

A repeated survey of rainforest birds was carried out along an elevation gradient on the slopes of Mt. Wilhelm in the Central Range of Papua New Guinea [29,30]. The study was completed along a 30 km long transect with eight sites, spanning from lowland floodplains at 200 m to the timberline at 3700 m. The sampling method was individual counts, which comprised surveying the bird communities at each site by point counts, mist-netting and random walks through the area. We assessed one categorical (trophic guild) and two continuous traits (body length and weight) of 238 species.

### Missing trait data simulations and data collection scenarios

**Fig 1:**
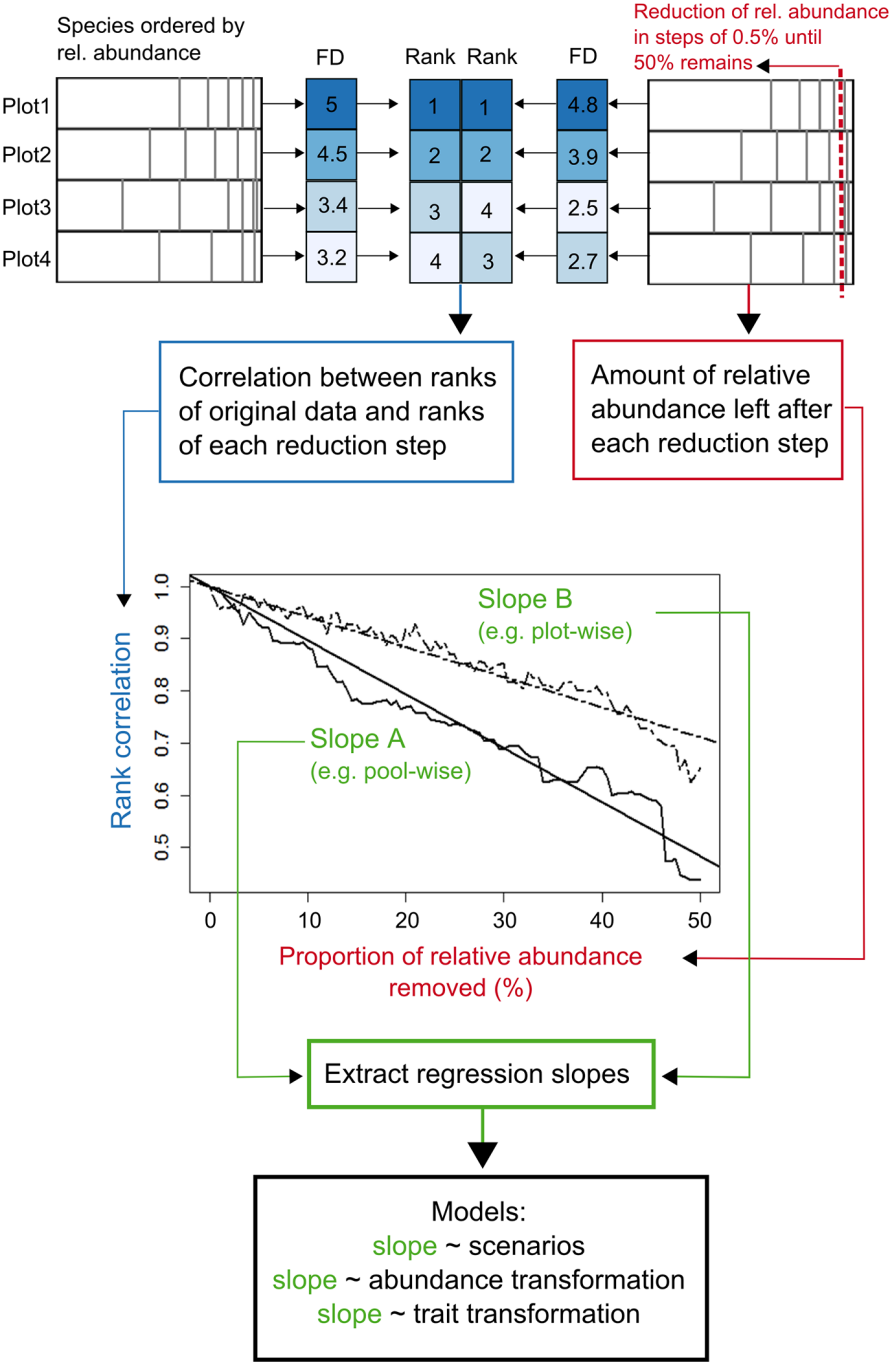
Flow diagram of the consecutive methodological steps. Upper left corner—in each plot species are ordered by their relative abundance and FD index is calculated for each plot of a community. Upper right corner—0.5% of the species relative abundance is removed in consecutive steps, starting with the least abundant species and FD index is then calculated again for each plot at each reduction step. Upper middle columns—plots are ranked based on the values of the FD index and the ranks of original data and data at each reduction step are correlated. Figure in the middle—regression slopes from fitting the linear model represent the robustness of FD index to missing trait data; in this example FD index is (A) less robust and (B) more robust to missing trait data (example RaoQ on head length of ants).

We simulated different degrees of trait data availability by progressively removing trait data, starting by omitting trait data for the least abundant species. Removing trait data generally results in some species not being included in the calculation of CWM and FD, i.e. part of the total community abundance is not considered. Removing the least abundant species first, mimics a frequently encountered sampling condition, where rarer species are often those with missing trait information [6]. We then followed the approach of Pakeman [6] to uniformly decrease the total abundance in a community by small steps (0.5%; Fig 1), in order to obtain a comparable continuous measure for hypothesis testing and comparison between communities. If a species accounts for less than 0.5% of total abundance, the whole species is removed in one step. If a species accounts for more than 0.5% of total abundance, the entire species is removed in several steps. The alternative to this approach is removing one species at a time, but this would result in unpredictable changes in relative abundance, since abundance distributions differ from community to community and from dataset to dataset. As an example, assume a plot with the following abundances (e.g. number of individuals), sorted by increasing abundance: {1, 2, 3, 4, 4, 7, 9, 14, 33, 89}. Expressed in relative abundances, rounded for simplicity to two decimals, this becomes {0.01, 0.01, 0.02, 0.02, 0.02, 0.04, 0.05, 0.08, 0.19, 0.54}. If we now remove 5% (i.e. 0.05) from that plot, we remove the first 3 species and 0.01 of the fourth species. In the second step, we would remove the remaining 0.01 of the fourth species, the fifth species, and 0.02 of the sixth species, and so forth for each step. Hence, for each of these steps we omit species, simulating that we do not consider them and their accompanied trait values when calculating measures of functional composition and diversity.

**Fig 2:**
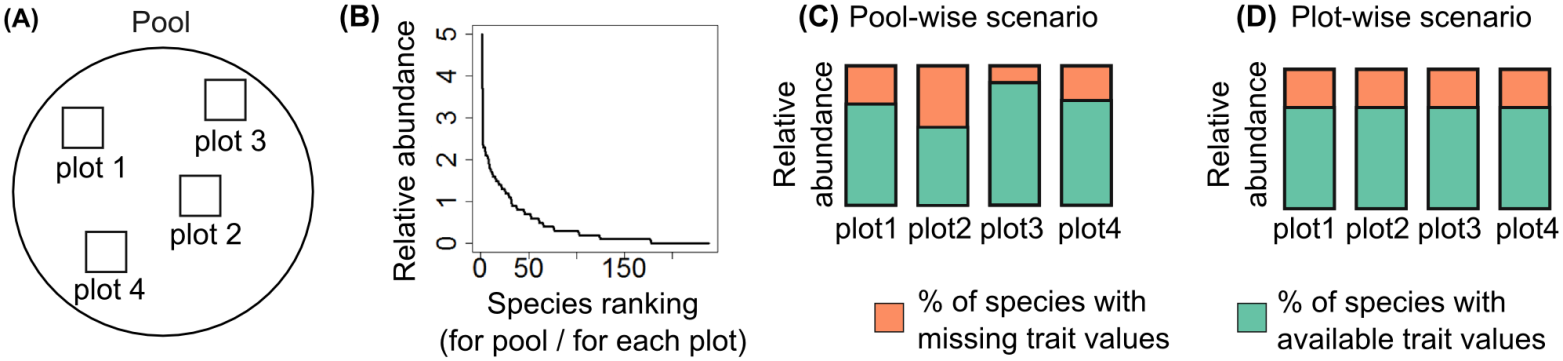
Plot-wise and pool-wise trait data thresholds. Schematic figure depicting plot-wise and pool-wise scenarios for setting the thresholds for trait data sampling. (A) species from all plots make up the pool of species; (B) species can be ordered by their abundance in each plot or in the whole pool; (C) the least abundant species in the whole pool of species are removed until reaching the desired threshold for trait sampling; (D) the least abundant species in each plot are removed until reaching the desired threshold for trait sampling.

As in Pakeman [6] we used two missing trait scenarios, pool-wise and plot-wise. For the pool-wise scenario (Fig 2), we first ranked species in the whole pool (i.e. species from all communities in a dataset) by abundance. We calculated the relative abundance of each species in the entire species pool and then we progressively removed the rarest species from the species pool, as demonstrated above. This resulted in differing availability of trait information between plots since some individual plots contain the removed species, and are affected, while others are not. Moreover, communities have different abundance structure (i.e. dominance-diversity curves) so that removing the same number of species in different communities can have different results. For the plot-wise scenario, we first ranked species in each plot by their abundance. We then calculated the relative abundance of each species for each plot and progressively removed the rarest species from each plot, as demonstrated above. In both scenarios, we removed 0.5% of the relative abundance at a time (Fig 1) until only 50% of the total abundance remained. We provide functions to apply this removal procedure to any community dataset in the R package "traitor" (see Data Accessibility for details).

### Transformation of abundance and trait data

We used different combinations of transformations of abundance and trait data for both scenarios (plot-wise and pool-wise, see previous section) in order to reveal how the two scenarios combined with different forms of data transformation influence the FD indices. For continuous traits we applied square root and log_10_ transformations (henceforth “log”). For abundance data we applied log (x + 1) and log (x / min(x) + 1) transformations where x is the abundance of a species and min(x) stands for the minimum positive abundance value of the species in the dataset. The log (x / min(x) + 1) transformation is a special case of a general form log (a × x + b), which is usually applied for data containing zeroes. Similar to the often used log (x + 1) it keeps the absence (i.e. original zero) to be zero after transformation, but unlike the log (x + 1) it also keeps the logarithmic character of the transformation when the values are rather low. For example, if the values are between zero and two, the character of log (x + 1) transformation is close to linear, whereas log (x / 0.1 + 1) keeps the typical log shape, therefore equalizing the species abundances. In the case of individual counts and frequency log (x + 1) equals log (x / min(x) + 1), therefore we calculated the latter only for the plant datasets with biomass and cover.

### Functional diversity indices

We assessed three FD indices using the *FD* package [31]. We first computed the trait dissimilarity matrices with the Gower distance for both single and multiple traits [32]. Then we calculated the FD indices: (1) functional richness (FRic), which reflects the range of functional trait variability in a given species assemblage; (2) functional evenness (FEve), which represents the evenness of abundance distribution across species traits; and (3) Rao’s quadratic entropy index (RaoQ), which captures the degree of divergence in the abundance distribution of species functional traits. In addition, we computed community weighted mean (CWM) which expresses the structure of trait values in the community. We computed all four indices for 100% of species and individuals in each plot and then repeated this approach (including recalculation of the Gower distance) for each reduction step.

### Robustness of FD indices to missing trait data

The values of FD indices calculated for each plot within a dataset can change when missing different amounts of trait data. As a result, plots can change in how functionally diverse they are relative to each other, ie. they may end up ranked in a different order by their values of FD indices (Fig 1). Here we assessed whether the ranking of FD values across plots was conserved when trait data were removed in order to evaluate how missing trait information would affect biological conclusions. To do this we calculated Spearman’s rank correlations between the FD index values of the original data (100% of species) and those of every step of the simulated reduction sequence (Fig 1). When the rank correlations are high, the order of samples according to their FD values is maintained between the original and the reduced data, even if FD values themselves change. In contrast to a focus on FD *per se* [7], using rank correlations allows us to assess how data availability could affect possible biological interpretations, as this represents the amount of biological information retained in the reduced datasets. We produced these simulations for each organism, FD index, sampling scenario (pool-wise and plot-wise), trait (separately and combined together), and abundance and trait transformation type.

We used linear regression models for combinations of all cases described in the previous section, where the log-transformed rank correlations were considered as a response variable and the amount of relative abundance remaining in the plot as an explanatory variable (as shown in the example in Fig 1). We then extracted slopes from each regression model in order to estimate the decline of rank correlations, and therefore robustness of FD indices to missing trait data, for all possible combinations of cases considered. The less negative a slope estimate was, the lower the decrease in rank correlations with data reduction, and therefore the more robust the FD index was to missing trait data. The intercept for these regressions was forced through 1 to account for the theoretical starting point of the slopes and to make the slopes comparable. In further analyses, we refer to the regression slopes fitted with linear models as "robustness", as it represents the rate of decline of rank correlations with missing trait values.

### Data analyses

#### Testing the effects of sampling scenarios, abundance distribution, abundance measures and their transformation

We tested which of the different predictors had a significant effect on the slope values extracted in the previous step, therefore indicating which variables affect the robustness of FD indices to missing trait data. We used linear mixed effects models with maximum likelihood estimation (*nlme* package, [33]) with regression slope values (from linear regression models described in previous section) as the response variable, and scenario (pool-wise or plot-wise), abundance transformation (transformation or no transformation), index (CWM, FRic, FEve, RaoQ), and their two-way and three-way interactions as fixed effects. Because the traits selected for each organism represent just a subset of all possible traits that can be measured, we used trait as a random factor in the linear mixed effect models. The models were performed for each organism type separately. In the case of plants, where three different abundance measures were applied (biomass, cover, and frequency) we also used the abundance measures (i.e biomass, cover, and frequency) as another fixed explanatory variable. Because we could not calculate slopes for all combinations of our explanatory variables (e.g. CWM of categorical traits) we used a mixed effects model, which allows an unbalanced design of the data [34].

#### Testing the effects of trait data structure and its transformation

In a separate model, we also tested the effect of trait transformation on the robustness of FD indices to missing trait values. For that we first calculated the skewness of all continuous trait distributions before and after each transformation type (square-root transformed, log_10_ transformed). Categorical traits were not included in this analysis. Trait values were always positively skewed. The difference in skewness between untransformed and transformed data indicated how much data transformation improved the normality of the traits. Small differences in skewness indicated that the trait was already close to normality and transformation was not necessary. We then tested with linear mixed effects models whether trait transformations improved the effect of missing trait data on the FD indices. In the models, regression slope values (from linear regression models described in the “Robustness of FD indices to missing trait data”) were used as the response variable and improvement in skewness, scenario (plot-wise or pool-wise), index (CWM, FRic, FEve, RaoQ) and their interactions were used as variables with fixed effects. Again, because traits considered in our analyses represent only a subset of all potential traits that can be measured for each organism, we used trait as a random factor in the models. The models were done for each organism type separately.

## Results

### The effect of missing trait data and the effect of sampling scenarios on FD indices

**Fig 3:**
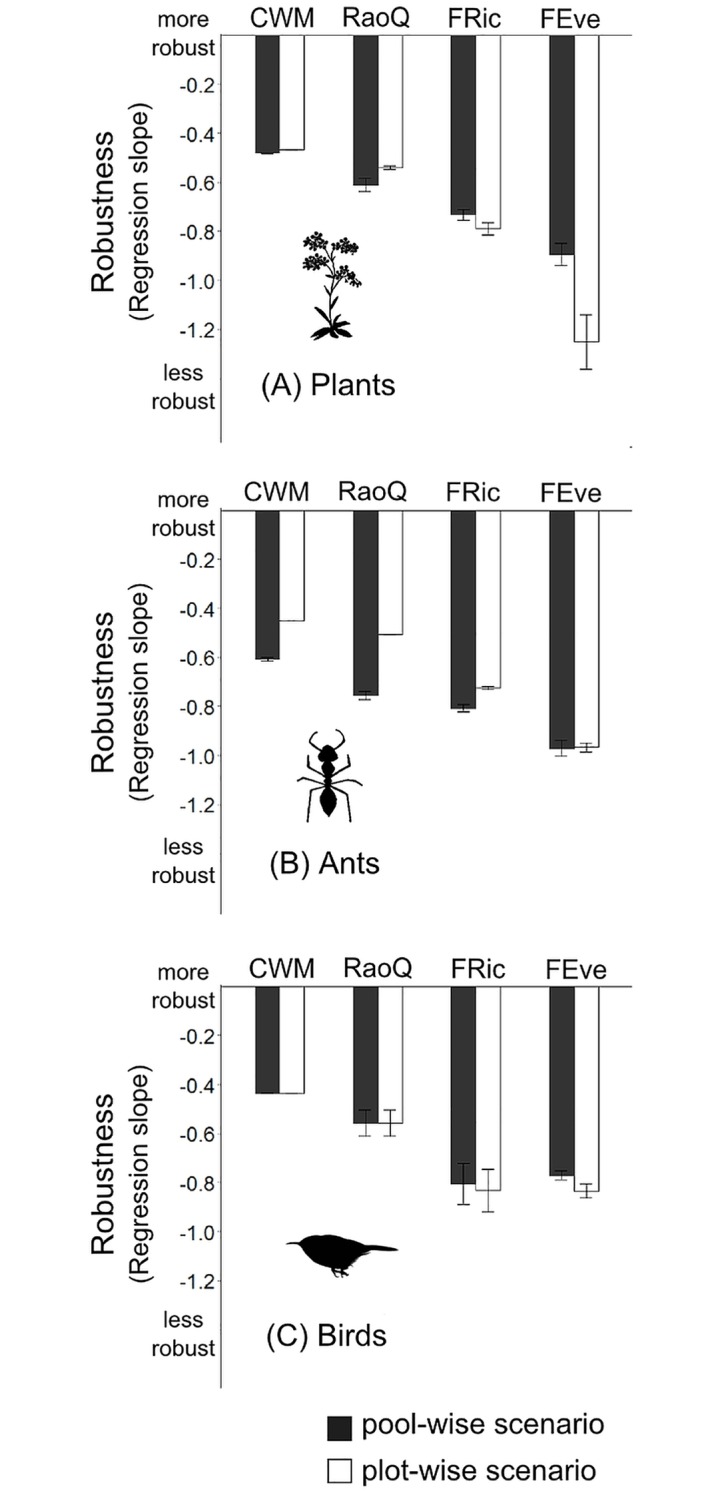
Effect of sampling scenario on FD index sensitivity. Barplots showing the results of linear mixed effects model, specifically the effect of the two sampling scenarios on the sensitivity of indices for three different types of organisms. The more negative the regression slope, the more sensitive the particular index is to missing trait information. The error bars denote the 95% confidence intervals. (A) plant community (n = 12 plots), (B) ant community (n = 58 plots), and (C) bird community (n = 8 plots).

All assessed indices were, as expected, sensitive to missing trait data (Table 1). Community weighted mean (CWM) was less sensitive to missing trait data than the FD indices for all three datasets. Within the FD indices Rao’s quadratic entropy index (RaoQ) was the least sensitive, followed by functional richness (FRic), and functional evenness (FEve) (Fig 3). The effects differed for the different community types. For plant data, FEve was more sensitive to missing trait information for the pool-wise scenario than for the plot-wise scenario. The other three indices were equally sensitive to missing trait information in both scenarios (Fig 3A). For the ant data, in the plot-wise scenario RaoQ was less sensitive to missing trait information, and CWM, FEve, and FRic were more similar in both sampling scenarios (Fig 3B). For the bird data, none of the interaction terms were statistically significant (Table 1), indicating that all indices were similarly sensitive to missing trait information for both scenarios (Fig 3C).

**Table 1:** Effect of sampling scenario and abundance transformation on FD index sensitivity.

Community	Predictors	Df	F	*P*
Plants	Scenario	1, 493	13.28	<0.001
	Index	3, 493	236.22	<0.001
	Abun.Transf.	1, 493	12.24	<0.001
	Scenario × Index	3, 493	21.10	<0.001
	Scenario × Abun.Transf.	1, 493	0.03	n.s.
	Index × Abun.Transf.	3, 493	27.20	<0.001
	Scenario × Index × Abun.Transf.	3, 493	1.66	n.s.
Ants	Scenario	1, 110	101.68	<0.001
	Index	3, 110	170.13	<0.001
	Abun.Transf.	1, 110	189.39	<0.001
	Scenario × Index	3, 110	24.84	<0.001
	Scenario × Abun.Transf.	1, 110	7.65	0.007
	Index × Abun.Transf.	3, 110	14.89	<0.001
	Scenario × Index × Abun.Transf.	3, 110	0.93	n.s.
Birds	Scenario	1, 84	0.29	n.s.
	Index	3, 84	20.25	<0.001
	Abun.Transf.	1, 84	0.48	n.s.
	Scenario × Index	3, 84	0.09	n.s.
	Scenario × Abun.Transf.	1, 84	0.005	n.s.
	Index × Abun.Transf.	3, 84	5.10	0.003
	Scenario × Index × Abun.Transf.	3, 84	0.09	n.s.

Results of general linear mixed effects models showing the effect of the two scenarios (pool-wise or plot-wise; "Scenario"), FD indices (functional richness, functional evenness, Rao’s Quadratic entropy index, and community weighted mean; "Index"), and abundance transformation ("Abun.Transf.") on robustness of FD indices to missing trait data (the regression slopes).

### The effect of abundance distribution, abundance measures and their transformation

**Fig 4:**
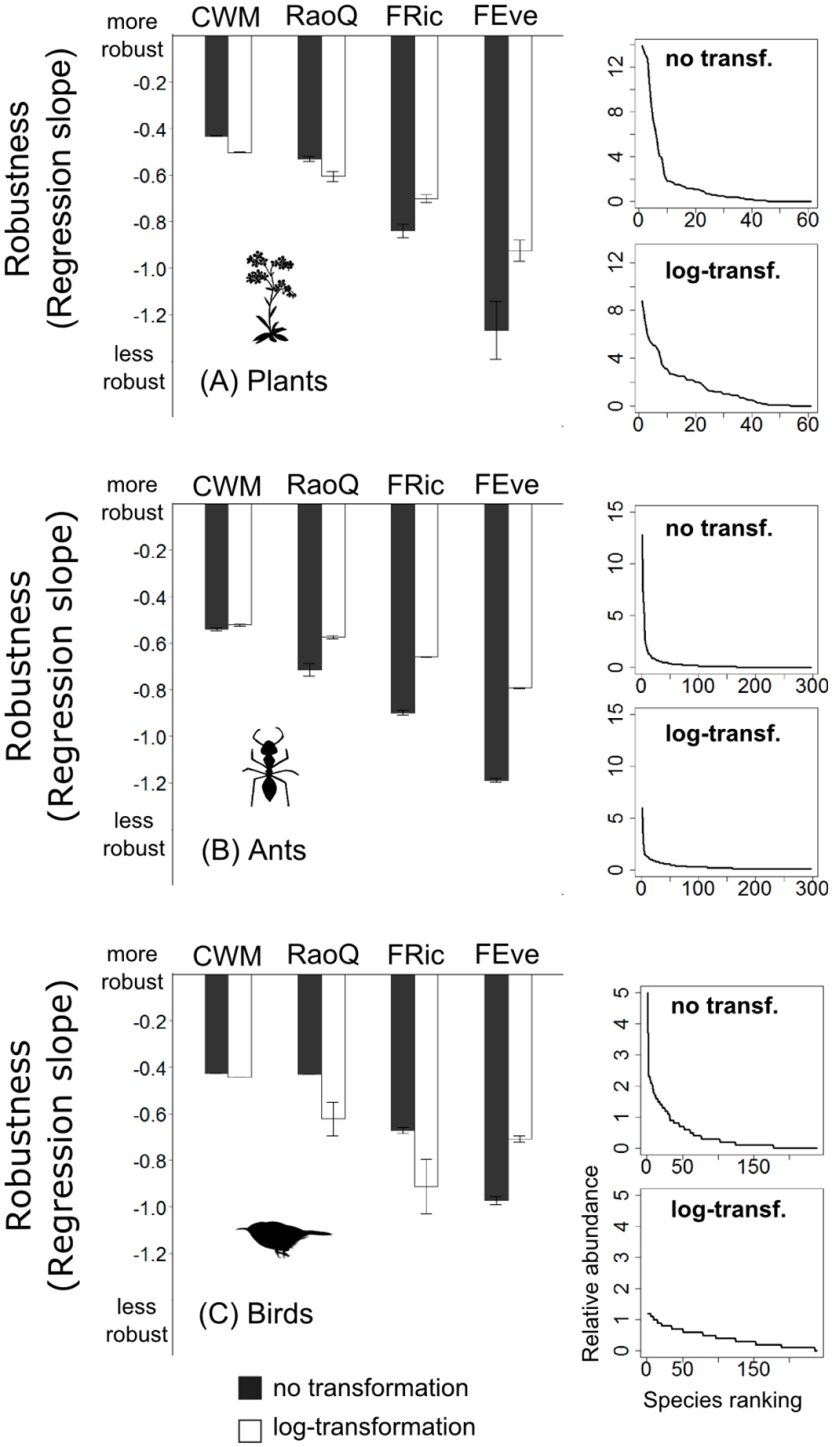
Effect of abundance transformation on FD index sensitivity. Barplots showing the results of linear mixed effects models, specifically the effect of the abundance transformation on the slopes for the three different types of organisms. The more negative the regression slope, the more sensitive the particular index is to missing trait information. The error bars denote the 95% confidence intervals. (A) plant community (n = 12 plots), (B) ant community (n = 58 plots), and (C) bird community (n = 8 plots). The right panels depict dominance-diversity curves for the respective organism dataset before and after log-transformation.

**Fig 5:**
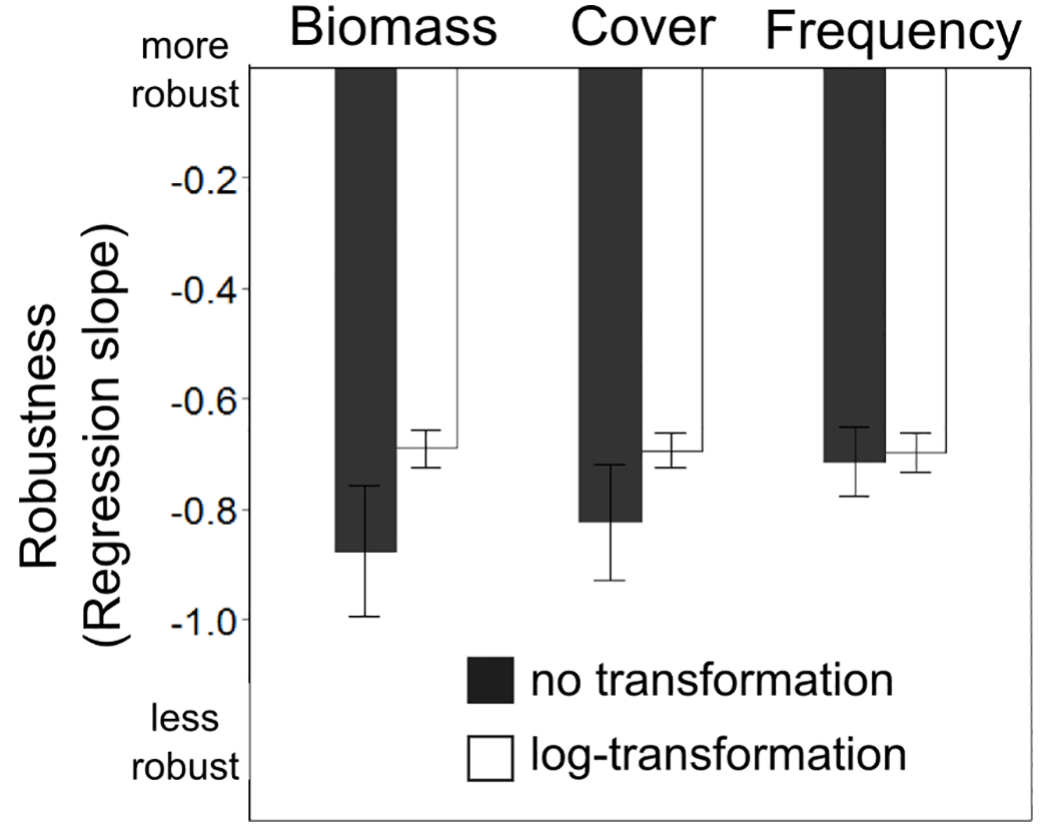
Effect of sampling method and abundance transformation on FD index sensitivity. Barplot depicting the results of linear mixed effects models, specifically the interaction between abundance transformation and the different abundance measures used in plant ecology (all three abundance measures were used for the same plant dataset in order to make their effects comparable). The effect of down-weighting the dominant species by log-transformation of their abundance was most pronounced in the biomass abundance measure. When log transformed, all three sampling methods have a very similar effect on the sensitivity of indices to missing trait data. Error bars denote the 95% confidence intervals.

For plant data, abundance transformation greatly decreased the sensitivity of FEve and FRic to missing trait data, but only slightly decreased RaoQ and CWM sensitivity (Fig 4A), which were however the least sensitive to missing trait data. When considering the three different abundance measures available for the plant dataset (biomass, frequency, and cover), the frequency measure was the least sensitive to missing trait data (Fig 5). The interaction between abundance transformation and abundance measure was significant (F_(2, 461)_ = 6.3; *P* = 0.002; see also S2 Appendix in Supplementary material); the differences between these measures were equalized after transformation (Fig 5). For ants, the interaction between abundance transformation and index indicated that FD indices computed from transformed species abundances were less sensitive to missing trait data than FD indices computed from original species abundances (Fig 4B). For birds, abundance transformation did not significantly change the sensitivity of FD indices (Fig 4C).

### The effect of trait distribution and transformation

**Fig 6:**
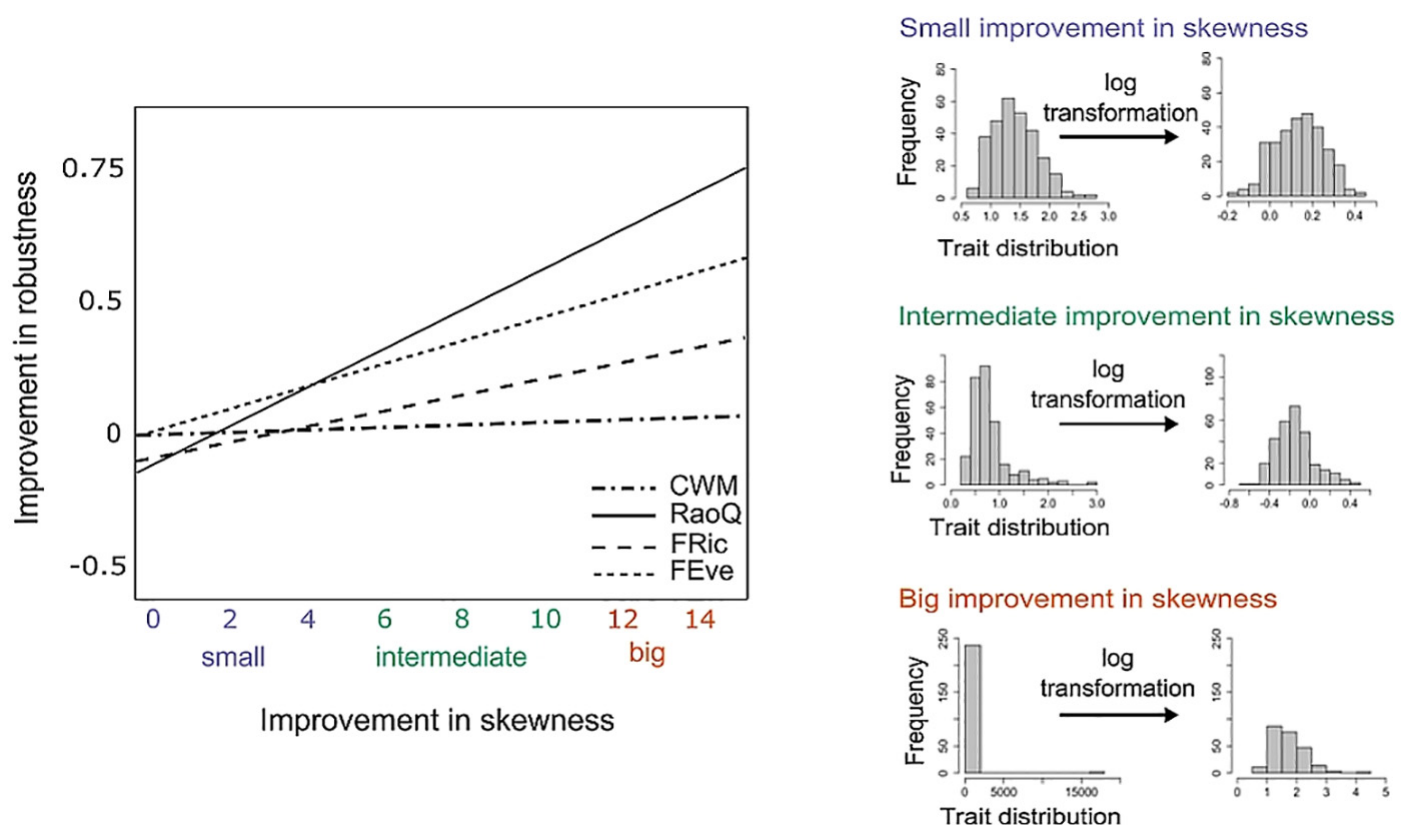
Effect of trait transformation on FD index sensitivity. The effect of trait transformation on the improvement in slope (transformed—untransformed trait data)—the bigger the improvement in slope, the more robust the index becomes to missing trait data (y axis). The right panels illustrate the different improvements in trait skewness, depicting examples of trait distribution before and after transformation, which correspond to the x axis of the main figure (matching colours).

The more normal the distribution of traits before transformation, the less sensitive the indices became to missing trait information (Fig 6). There was a significant interaction between the change in skewness (from untransformed to log-transformed trait data) and index (F_(3, 274)_ = 6.78; *P* > 0.001; see also S2 Appendix in Supplementary material). Trait data transformation most improved the accuracy of RaoQ, followed by FEve, FRic, and there was no improvement for CWM, as expected since trait transformation did not alter the ranking in species trait values (Fig 6).

## Discussion

### The effect of missing trait data and the effect of sampling scenarios on FD indices

The number of species for which trait information is available can influence not only the accuracy of functional diversity indices [7], but also the possible biological interpretation. We demonstrated this by showing that the ranking of FD values changes across communities with an increasing amount of missing trait data compared to a complete dataset. Our results also confirmed the previous finding that some FD indices are more sensitive than others to missing trait information [7,11]. FD indices were not as robust as CWM, which is heavily influenced by the most abundant species. Functional evenness was the most sensitive index to missing data, perhaps because the missing trait information for rare species removed extreme or outlier trait values.

Our results suggest that neither pool-wise nor plot-wise scenario should ultimately be considered as "best" when setting data completeness thresholds, and that the decision is contingent upon the particular study system, FD index, and research question. Previous studies have suggested that plot-wise sampling is more appropriate because it produces values closest to the FD values obtained with a complete dataset [7] and can be performed with less sampling effort [14]. However, our approach of testing the ranking of FD rather than the actual values showed no consistent trend in this direction. When considering the effect of missing data under the two sampling scenarios indices behaved differently between the different communities.

### The effect of abundance distribution, abundance measures and their transformation

Abundance distribution, abundance measures (biomass, cover estimate, frequency) and abundance transformation influenced the robustness of FD indices to the amount of missing trait information. Transforming the abundance leads to a shift of the abundance distribution in a community, thereby changing the relative abundance. In our simulation approach this implies that fewer species are removed at a given level of relative abundance compared to untransformed data. The different indices are designed to capture different aspects of diversity, and they thus responded differently to transformation. We found that functional evenness always improved with transformation because species were given more equal weighting, but functional divergence (RaoQ) improved less or not at all since transformation essentially shrinks the range of values. CWM remained unaffected as it focuses on the dominants even before transformation. Care should be taken in studies comparing functional diversity between communities with different abundance distribution and with the use of different FD indices, since transformation may have different effects on the accuracy of FD indices.

Abundance transformation should not be considered as a purely technical matter, because it reflects our understanding of the community structure. Transforming abundance changes the community structure by flattening the dominance-diversity curves [17]. And therefore the question whether to transform abundance mainly depends on the biological question behind the particular analysis. If, for example, we followed the mass-ratio hypothesis [8] focusing on the effect of the dominant species in a community, then dominant species should be given more weight, in which case abundance transformation would not be the most appropriate option. On the other hand, if the scope of our study included coexistence mechanisms, abundance transformation could better reveal the structure of our community giving biological relevance not only to the few dominant species.

Different abundance measures also influence the number of species for which traits are needed in order to achieve a desired amount of information for a given community. This trend is visible when we consider three different abundance measures available for the plant community in our study (biomass, cover, frequency). For example, the biomass measure after transformation resembled the frequency measure without transformation. The difference between the abundance measures can be explained by how evenly spread the abundance values are [35,36]. Using plant biomass as a measure typically produces the most uneven abundance values. In our plant community for example, the least abundant species accounted for as little as 0.001 g (generally, the precision of weighing), and the dominants exceeded 10g, so the potential difference is several orders of magnitude. Frequency, on the other hand, will have smaller differences, as in our data where it was measured in 25 subplots, and so the possible maximum was only 25 fold higher than the possible minimum. Transforming the abundance data therefore equalizes the effects of different abundance measures, which can be used as a way of standardization in comparative studies and meta-analyses.

### The effect of trait distribution and transformation

Our results show that the more the transformation improves the trait data normality (i.e. from very skewed to normal), the more it improves the robustness of the FD indices to missing trait information. FD indices were sensitive to changes in the distribution of trait values (see also [20]), as the changes in distribution altered the functional differences between species in the communities. This was true mainly for indices that comprise the variance of trait distribution, i.e. functional richness and functional divergence (RaoQ). CWM, on the other hand, focuses on the dominant species, and therefore its robustness to missing trait information was not affected by trait transformation (e.g., big species are still big species after transformation).

Trait distribution is of a great importance, as trait data in natural communities vary in the extent that they deviate from the normal distribution, which also influences FD calculations [19,20]. Outlying values or values spreading across several orders of magnitude manifest as the skewness of the data. Some traits are by their nature more normally distributed in a community (e.g. specific leaf area in plants), whereas some are usually highly skewed (e.g. seed weight in plants, body size in ants and body weight in birds; Fig 6 "Big improvement in skewness") and are therefore routinely transformed to meet normality assumptions. As the data are on a ratio scale, the outliers are usually positive, and so the high skewness means that there are either more positive outliers, or there is one highly positive outlier. The most extreme case is when a species with the most different trait value is the rarest one. An example can be found in each of our communities: (1) the plant dataset is dominated by small-seeded grasses and forbs but has a big-seeded and rare legume species (*Lathyrus pratensis* L.), (2) the ant dataset is dominated by small species but the giant forest ant (*Camponotus gigas* Latreille) is very rare, and (3) the bird dataset is dominated by small flying species but also contains the much larger and flightless cassowary (*Casuarius bennetti* Gould, 1857), which is very rare.

Our results empirically support previous suggestions that trait values should be log-transformed, as the relationship between any two species is best characterized by the difference in logs, i.e. by the ratio of traits, rather than by the absolute difference between them [21,22]. Like abundance transformation, the choice of transforming the traits is also dependent on the particular research question. When upscaling for the questions regarding ecosystem functioning, the absolute values of a trait are more important. For example, sometimes it is more interesting to know strictly how tall a species is (absolute value of a trait), rather than how tall a species is compared to its neighbours (transformed value of a trait). On the other hand, it is important to transform trait data when detecting the processes behind community assembly and/or species co-existence, for example how tall a species is compared to its neighbours.

### Practical implications

Since functional diversity indices are sensitive to missing trait data, and this sensitivity is further affected by abundance structure, different abundance measures and the transformation of abundance and trait data, all should be given careful consideration during experimental design, analyses and interpretation of functional diversity. One can obtain trait values for all species in the desired study system by: (1) measuring the traits, (2) using trait data from trait databases available, and/or (3) inferring trait data from phylogeny [12]. Even with these tools it is often not possible to have trait data for all species in a community, due to limited time and resources, incomplete or unavailable trait databases and/or constraints of imputation approaches. In this case, one needs to work with a number of traits that are feasible to measure and to make an *a priori* decision for how many and for which species the trait data is essential and whether to set this particular missing trait data safety threshold for the whole pool of species or per each plot separately. As shown and discussed above, in some cases abundance and/or trait transformation can increase the robustness of FD indices, which in practical terms means that for a given amount of effort one could increase the amount of replication of sampling for species abundances at the expense of measuring traits.

To assist researchers in assessing effects of missing trait data and planning trait sampling campaigns we provide the R package "traitor" (see Data Accessibility). The functions in this package help ecologists to estimate trait data availability for their datasets and provide a list of species for which traits need to be obtained to increase data availability and as a result improve the accuracy of FD calculations. Given an existing community data set and information for which species trait data is available, the function within the package estimates how much of the relative species abundance is covered by the available trait data. This is either done on a pool-wise (across all plots) or plot-wise (for each single plot) basis to account for our findings that neither the pool-wise nor plot-wise scenario produce systematically less reliable results. Additionally, the package contains functions to assess how omitting trait data in a given data set can bias FD measures, i.e. it provides the tools to conduct the analyses in this study, and those in [11] and [7]. It can also be used to assess the impact of species loss on functional diversity, i.e. the vulnerability of communities to the extinction of rare species (e.g. [23]).

## Conclusions

Our study demonstrates that not only the amount of trait data available, but also the species abundance structure and distribution of trait values have a significant effect on the calculation of FD indices. Consequently, their transformation greatly affects the evaluation of functional diversity. Even though the details about data structure and its transformation often appear a trivial part of FD analyses, we show that they are as important as the amount of available species trait information. Thus the careful treatment of both abundance and trait data is essential to interpret functional diversity and can, to a certain degree, even compensate for the lack of trait data. Such methodological choices are crucial for a faithful evaluation of functional diversity.

## Supporting Information

S1 Appendix Study sites and sampling methods. Detailed description of the sampling and trait collection in the three communities.
(DOCX)

S2 Appendix Results of the linear mixed effect models. Tables A1 –A5 presenting results of all linear mixed effects models.
(DOCX)

S1 Dataset Data used for the analysis. Abundance and trait data for our plant, ant, and bird communities.
(ZIP)
