# Correction: Evaluating Functional Diversity: Missing Trait Data and the Importance of Species Abundance Structure and Data Transformation

**DOI:** 10.1371/journal.pone.0152532

**Published:** 2016-03-23

**Authors:** The PLOS ONE Staff

The following information is missing from the Funding section: NSP and MW were funded by Faculty of Science of the University of South Bohemia.

The affiliation for the seventh author is incorrect. Matthias Weiss is affiliated with Institute of Entomology, Biology Centre CAS, České Budějovice, Czech Republic, and Department of Zoology, Faculty of Science, University of South Bohemia, České Budějovice, Czech Republic.

There is an error in affiliation 14 for author Francesco de Bello in the PDF. The publisher apologizes for the error. Affiliation 14 should be: Institute of Botany, Biology Centre CAS, Třeboň, Czech Republic.
