# Supplementary material for: Evaluating Functional Diversity: Missing Trait Data and the Importance of Species Abundance Structure and Data Transformation
Source: PLoS One. 2016 Feb 16;11(2):e0149270. doi: 10.1371/journal.pone.0149270 (PMC4755658; doi:10.1371/journal.pone.0149270)
Supplement: S1 Appendix — Detailed description of the sampling and trait collection in the three communities. (DOCX) [file pone.0149270.s001.docx]

Supplementary materials for: Májeková, M. et al. Evaluating functional diversity: missing trait data and the importance of species abundance structure and data transformation.

– PLOS One.

**S1 Appendix: Detailed description of the study systems**

**Plant data**

Plant data consists of 12 plots and 62 species. Plants were surveyed in a species-rich wet meadow located 10 km south-east of České Budějovice, Czech Republic (48°57’N, 14°36’E, altitude 510 m). Plants were surveyed in June 2006 as a part of a long term experiment (1994-2014). The plant community is an oligotrophic wet species-rich meadow (around 30 spp. of vascular plants per m^2^) dominated by a tussock grass, *Molinia caerulea* (L.) Moench*.* Other more abundant species included mostly grasses and over 10 species of sedges (*Carex* L. spp.). The meadow, as those in the region around, has been traditionally extensively mown for centuries.

In 1994, 12 permanent mown plots were established, each 2 × 2 m. Fertilization and dominant removal were applied as treatments in a factorial design. The four treatment combinations (fertilization and removal; fertilization and no removal; no fertilization and removal; no treatment) were repeated three times. The fertilization treatment consisted of annual application of a commercial NPK fertilizer (12% N, as nitrate and ammonium, 19% P, as P_2_O_5_, and 19% K, as K_2_O). *Molinia caerulea* tillers were manually removed with a screwdriver in April 1995 with a minimum of soil disturbance and new individuals of *M. caerulea* were removed annually. More details about the experimental setup are given in ([1–3].

We chose traits that represent the L-H-S scheme proposed by Westoby (1998)[4]: specific leaf area (SLA), plant height, and seed mass. These traits represent independent dimensions of plant ecological strategies [4–7]. We measured plant height, specific leaf area and seed mass values for 42 species following protocols in [8] for a minimum of 10 individuals collected in the different treatments where the species occurred [9]. Trait values were then averaged to allow comparisons with values taken from LEDA trait database [10] for the remaining species (approximately 20 depending on the sampling method). Specific leaf area represents the ratio of leaf area to dry weight (m^2^ kg^-1^). The two categorical traits, growth form and position of leaves along the stem, were also taken from LEDA trait database and from local floras.

**Ant data**

Ant data consists of 59 plots and 297 species. Ant data were collected in April and May 2010 as part of the Stability of Altered Forest Ecosystems (SAFE) project in Sabah, Malaysia [11,12]. In total the survey included 59 plots in different habitat types; 9 plots in a single block of oil palm, 32 plots in two blocks of secondary forest and 18 plots in two blocks of primary forest. Ant sampling was conducted at 2^nd^ order survey points arranged in equilateral triangles with sides of 178 m. Oil palm and secondary forest sites are located in Benta Wawasan, a matrix of oil palm *Elaeis guineensis* Jacq. monocultures planted in the year 2000 and twice-logged forest of varied quality. Primary forest sites are in Maliau Basin Conservation area, and not subject to anthropogenic disturbance.

For each sampling point, as close to the 2^nd^ order point as possible, a 4 × 4 m quadrat was set out. In each square meter of the quadrat a 12 × 12 cm soil sample with a depth of 10 cm was extracted (16 soil pits per quadrat) and the soil searched for ants for 10 person-minutes. In addition, dead wood within the quadrat was examined for ants by removing the bark and investigating any holes or crevices. All specimens were preserved in 80% ethanol. Ants were identified to species level using published keys and online resources (see references in [13]), with the majority of specimens being assigned to morphospecies due the inaccessibility of a reference collection and lack of adequate keys for certain rare or hyperdiverse Bornean genera.

Morphological traits related to the species’ ecology were measured to the nearest 0.01 mm using an ocular micrometer attached to a Lecia MS5 stereo-microscope (Leica Microsystems, Heerbrugg, Switzerland). The following traits were measured for the worker caste only: head length, the ratio of leg length to body size, pilosity, sculpturing. Since the reproductive castes have a non-foraging role and major castes are not sampled effectively using the above protocol they were not included in the study. Head length is highly correlated, and thus a good surrogate of body length, which relates to many life history traits such as prey selection [14] and thermal tolerance [15]. Leg length may be an adaptation to carrying different loads of foraging items [16], and the spatial niche in which the species forages [17]. As it is highly correlated with body size we chose to express leg length as a ratio of body size: the sum of the hind femur and hind tibia length/head length. Pilosity and sculpturing may be related to the ant thermal tolerance. See [18]for a summary of ant traits and their hypothesised ecological relevance.

**Bird data**

Bird data comprise 8 plots and 238 species. A repeated survey of forest birds was carried out along an elevational rainforest gradient on the slopes of Mt. Wilhelm (4509 m a.s.l.) in the Central Range of Papua New Guinea between 9^th^ April 2010 and 15^th^ October 2012 [19,20]. The study was completed along a 30 km long transect with eight study sites, spanning from lowland floodplains of the Ramu river (200 m) to the timberline at 3700 m, evenly spaced at 500 m elevational increments. At each elevation, bird communities were surveyed by point counts (n=14), mist-netting (n=11) and random walks (20 hrs total) through the area (80 ha). Point counts were conducted at 16 points regularly spaced along a 2250 m transects (successive points 150 ± 5 m apart to avoid overlap). Transects at each site were directed through the representative and diverse microhabitats in the area (e.g. ridges, valleys, streams). All birds seen or heard within 50 m of the point were recorded. Censuses began 15 min before sunrise, at a randomly selected point, and each count lasted 15 min with all 16 points surveyed before 11am. At each study site, we mist-netted birds along a 200 m line (using nets 2.5 m high × 12-18 m long, mesh 16 mm) from 5:30am till 5:30pm daily. We identified all mist-netted birds, marked them with colour bands, and released them within 10 min. Finally, we walked along existing trails and along point-count transects, surveying the area of 80 ha evenly, and noted all species identified during these random walks (within a 50 m radius). Walks lasted 2-3 h per day (starting at 3pm, walking distance = 3-4 km/d), and were standardized to 20 h per site. All surveys were conducted by three people, all of whom had previous experience with bird surveys in Papua New Guinea. We also recorded vocalizations at each survey point during surveys and any unrecognized bird vocalizations during our random walks (using a Marantz PMD 620 recorder and a Seinnheiser ME67 microphone) to allow later identification.

Birds were identified using the species-level taxonomy of Handbook of the birds of the world [21] and updated according to IOC Bird World List 5.1. All species were categorized into three broad trophic guilds: insectivores, herbivores (granivores + frugivores) and omnivores (with equal intake of different items), based on dietary information from the field and in standard references [19, 21, 22, 23]. Only forest species were included in the analyses and all raptors and swifts were excluded (68 individuals of 15 species) since it was difficult to sample them in a standardized manner from the forest interior. We assessed three ecologically relevant traits; one categorical (trophic guild) and two continuous traits (body length and weight) of 238 species. The body measurements (body length and weight) for most of the birds in analysis (238 species, 87.5% of total) were obtained as averages from mist-netted birds. Data on body measurements for the other 34 bird species were obtained from Museum collections (Natural History Museum of Denmark, Queensland museum) and from literature [21].

**References**

1. Lepš J. Nutrient status, disturbance and competition: an experimental test of relationships in a wet meadow. J Veg Sci. Spie; 1999;10: 219–230.

2. Leps J. Variability in population and community biomass in a grassland community affected by environmental productivity and diversity. 2004;1.

3. Lepš J. Scale- and time-dependent effects of fertilization, mowing and dominant removal on a grassland community during a 15-year experiment. J Appl Ecol. 2014;51: 978–987.

4. Westoby M. A leaf-height-seed (LHS) plant ecology strategy scheme. Plant Soil. 1998;199: 213–227.

5. Westoby M, Falster DS, Moles AT, Vesk PA, Wright IJ. Plant Ecological Strategies: Some Leading Dimensions of Variation Between Species. Annu Rev Ecol Syst. 2002;33: 125–159.

6. Reich PB. The world-wide “fast–slow” plant economics spectrum: a traits manifesto. J Ecol. 2014;102: 275–301.

7. Laughlin DC. The intrinsic dimensionality of plant traits and its relevance to community assembly. J Ecol. 2014;102: 186–193.

8. Cornelissen JHCA, Lavorel SB, Garnier EB, Díaz SC, Buchmann ND, Gurvich DEC, et al. A handbook of protocols for standardised and easy measurement of plant functional traits worldwide. 2003; 335–380.

9. Lepš J, de Bello F, Šmilauer P, Doležal J. Community trait response to environment: disentangling species turnover vs intraspecific trait variability effects. Ecography (Cop). 2011;34: 856–863.

10. Kleyer M, Bekker RM, Knevel IC, Bakker JP, Thompson K, Sonnenschein M, et al. The LEDA Traitbase: a database of life-history traits of the Northwest European flora. J Ecol. 2008;96: 1266–1274.

11. Ewers RM, Didham RK, Fahrig L, Ferraz G, Hector A, Holt RD, et al. A large-scale forest fragmentation experiment : the Stability of Altered Forest Ecosystems Project. Philos Trans R Soc B Biol Sci. 2011;366: 3292–3302. doi:10.1098/rstb.2011.0049

12. Luke SH, Fayle TM, Eggleton P, Turner EC, Davies RG. Functional structure of ant and termite assemblages in old growth forest, logged forest and oil palm plantation in Malaysian Borneo. Biodivers Conserv. 2014;23: 2817–2832.

13. Bishop TR. Functional diversity and community assembly patterns in ant ( Hymenoptera : Formicidae ) communities across a forest disturbance gradient in Sabah , Malaysia. 2012.

14. Traniello JFA. Comparative foraging ecology of north temperate ants: The role of worker size and cooperative foraging in prey selection. Insectes Soc. 1987;34: 118–130.

15. Heinze J, Foitzik S, Kipyatkov VE, Lopatina E. Latitudinal variation in cold hardiness and body size in the boreal ant species Leptothorax acervorum (Hymenoptera:Formicidae). Entomol Gen. 1998;22: 305–312.

16. Feener D. H. J, Lighton JRB, Bartholomew G a. Curvilinear Allometry, Energetics and Foraging Ecology: a Comparison of Leaf-Cutting Ants and Army Ants. Funct Ecol. 1988;2: 509–520.

17. Kaspari M, Weiser MD. The size-grain hypothesis and interspecific scaling in ants. Funct Ecol. 1999;13: 530–538.

18. Gibb H, Stoklosa J, Warton DI, Brown AM, Andrew NR, Cunningham SA. Does morphology predict trophic position and habitat use of ant species and assemblages? Oecologia. 2015;177: 519–531.

19. Tvardíková K. Trophic relationships between insectivorous birds and insect in Papua New Guinea. University of South Bohemia. 2013.

20. Sam K, Koane B. New avian records along the elevational gradient of Mt. Wilhelm, Papua New Guinea. Bull Br Ornithol Club. 2014; 116–133.

21. Del Hoyo, J. et al. (1992–2011). Handbook of the Birds of the World. – LynxEdictions.

22. Beehler, B. M., Pratt, T. K., Zimmerman, D. A., Bell, H. L., Finch, B. W., Diamond, J. M., & Coe, J. (1986). Birds of New Guinea (No. 9). Princeton: Princeton University Press

23. Pratt, T. K. and Beehler B. B. (2014). Birds of New Guinea. – Princeton University Press.
