# Supplementary material for: Evaluating Functional Diversity: Missing Trait Data and the Importance of Species Abundance Structure and Data Transformation
Source: PLoS One. 2016 Feb 16;11(2):e0149270. doi: 10.1371/journal.pone.0149270 (PMC4755658; doi:10.1371/journal.pone.0149270)
Supplement: S2 Appendix — Tables A1 –A5 presenting results of all linear mixed effects models. (DOCX) [file pone.0149270.s002.docx]

Supplementary materials for: Májeková, M. et al.. Evaluating functional diversity: missing trait data and the importance of species abundance structure and data transformation.

– PLOS One

**S2 Appendix: Results of the linear mixed effect models**

Table A1. Full results of the general linear mixed effects models for plant data showing the effect of the two scenarios (pool-wise and plot-wise; “Scenario”), FD indices (functional richness, functional evenness, Rao’s Quadratic entropy index, and community weighted mean; “Index”), and abundance transformation (“Abun.Transf.”) on robustness of FD indices to missing trait data (the regression slopes).

| Predictors | Df | F | *P* |
| --- | --- | --- | --- |
| Intercept | 1, 493 | 110.8 | <0.001 |
| Scenario | 1, 493 | 13.28 | <0.001 |
| Index | 3, 493 | 236.22 | <0.001 |
| Abun.Transf. | 1, 493 | 12.24 | <0.001 |
| Scenario × Index | 3, 493 | 21.10 | <0.001 |
| Scenario × Abun.Transf. | 1, 493 | 0.03 | n.s. |
| Index × Abun.Transf. | 3, 493 | 27.20 | <0.001 |
| Scenario × Index × Abun.Transf. | 3, 493 | 1.66 | n.s. |

Table A2. Full results of the general linear mixed effects models for ant data showing the effect of the two scenarios (pool-wise and plot-wise; “Scenario”), FD indices (functional richness, functional evenness, Rao’s Quadratic entropy index, and community weighted mean; “Index”), and abundance transformation (“Abun.Transf.”) on robustness of FD indices to missing trait data (the regression slopes).

| Predictors | Df | F | *P* |
| --- | --- | --- | --- |
| Intercept | 1, 110 | 311.7 | <0.001 |
| Scenario | 1, 110 | 101.68 | <0.001 |
| Index | 3, 110 | 170.13 | <0.001 |
| Abun.Transf. | 1, 110 | 189.39 | <0.001 |
| Scenario × Index | 3, 110 | 24.84 | <0.001 |
| Scenario × Abun.Transf. | 1, 110 | 7.65 | 0.007 |
| Index × Abun.Transf. | 3, 110 | 14.89 | <0.001 |
| Scenario × Index × Abun.Transf. | 3, 110 | 0.93 | n.s. |

Table A3. Full results of the general linear mixed effects models for bird data showing the effect of the two scenarios (pool-wise and plot-wise; “Scenario”), FD indices (functional richness, functional evenness, Rao’s Quadratic entropy index, and community weighted mean; “Index”), and abundance transformation (“Abun.Transf.”) on robustness of FD indices to missing trait data (the regression slopes).

| Predictors | Df | F | *P* |
| --- | --- | --- | --- |
| Intercept | 1, 84 | 37.1 | <0.001 |
| Scenario | 1, 84 | 0.29 | n.s. |
| Index | 3, 84 | 20.25 | <0.001 |
| Abun.Transf. | 1, 84 | 0.48 | n.s. |
| Scenario × Index | 3, 84 | 0.09 | n.s. |
| Scenario × Abun.Transf. | 1, 84 | 0.005 | n.s. |
| Index × Abun.Transf. | 3, 84 | 5.10 | 0.003 |
| Scenario × Index × Abun.Transf. | 3, 84 | 0.09 | n.s. |

Table A4. Full results of the general linear mixed effects models for plant data showing the effect of the two scenarios (pool-wise and plot-wise; “Scenario”), FD indices (functional richness, functional evenness, Rao’s Quadratic entropy index, and community weighted mean; “Index”), abundance transformation (“Abun.Transf.”), and abundance measure (“Measure”) on robustness of FD indices to missing trait data (the regression slopes).

| Predictors | Df | F | *P* |
| --- | --- | --- | --- |
| Intercept | 1, 461 | 104.1 | <0.001 |
| Scenario | 1, 461 | 14.1 | <0.001 |
| Index | 3, 461 | 251.6 | <0.001 |
| Abun.Transf. | 1, 461 | 13.1 | <0.001 |
| Measure | 2, 461 | 3.8 | 0.02 |
| Scenario × Index | 3, 461 | 22.5 | <0.001 |
| Scenario × Abun.Transf. | 1, 461 | 0.03 | n.s. |
| Scenario × Measure | 2, 461 | 0.5 | n.s. |
| Index × Abun.Transf. | 3, 461 | 28.9 | <0.001 |
| Index × Measure | 6, 461 | 2.8 | 0.01 |
| Measure × Abun.Transf. | 2, 461 | 6.3 | 0.002 |
| Scenario × Index × Abun.Tranf. | 3, 461 | 1.7 | n.s. |
| Scenario × Index × Measure | 6, 461 | 2.3 | 0.04 |
| Scenario × Abun.Transf. × Measure | 2, 461 | 2.1 | n.s. |
| Index × Abun.Transf. × Measure | 6, 461 | 1.2 | n.s. |
| Scenario × Index × Abun.Transf. × Measure | 6, 461 | 0.2 | n.s. |

Table A5. Full results of the general linear mixed effects models showing the effect of trait data transformation (“Trait Transf.”), difference in skewness of trait data before and after trasformation (“Skewness”), the two scenarios (pool-wise and plot-wise; “Scenario”), and FD indices (functional richness, functional evenness, Rao’s Quadratic entropy index, and community weighted mean; “Index”) on robustness of FD indices to missing trait data (the regression slopes).

| Predictors | Df | F | *P* |
| --- | --- | --- | --- |
| Intercept | 1, 274 | 12.0 | 0.001 |
| Scenario | 1, 274 | 0.6 | n.s. |
| Index | 3, 274 | 3.5 | 0.02 |
| Trait | 1, 274 | 1.1 | n.s. |
| Skewness | 1, 274 | 47.4 | <0.001 |
| Scenario × Index | 3, 274 | 0.1 | n.s. |
| Scenario × Trait | 1, 274 | 0.3 | n.s. |
| Scenario × Skewness | 1, 274 | 2.0 | n.s. |
| Index × Trait | 3, 274 | 1.3 | n.s. |
| Index × Skewness | 3, 274 | 6.8 | <0.001 |
| Trait × Skewness | 1, 274 | 1.8 | n.s. |
| Scenario × Index × Trait | 3, 274 | 0.1 | n.s. |
| Scenario × Index × Skewness | 3, 274 | 1.8 | n.s. |
| Scenario × Trait × Skewness | 1, 274 | <0.01 | n.s. |
| Index × Trait × Skewness | 3, 274 | 1.3 | n.s. |
| Scenario × Index × Trait × Skewness | 3, 274 | 0.1 | n.s. |
